# Imaging through scattering media with the auxiliary of a known reference object

**DOI:** 10.1038/s41598-018-27754-x

**Published:** 2018-06-25

**Authors:** Wanqin Yang, Guowei Li, Guohai Situ

**Affiliations:** 1 Shanghai Institute of Optics and Fine Mechanics, Chinese Academy of Sciences, Shanghai, 201800 China; 2 University of Chinese Academy of Sciences, Beijing, 100049 China

## Abstract

Imaging through scattering media has been one of the main challenges in optics, and are encountered in many different disciplines of sciences, ranging from biology, mesoscopic physics to astronomy. Recently, various methods have been proposed. In this manuscript, we propose a robust method for imaging through scattering media in a reflective geometry, a scenario widely encountered in non-invasive and marker-free biological imaging. The proposed method relies on the a priori information of a known reference object in the neighborhood of the target, and uses it as an auxiliary to reconstruct the target image. We show that the target image can be analytically reconstructed from the autocorrelation of the recorded speckle if the reference is point-like, otherwise, deconvolution with the reference speckle should be performed. We experimentally demonstrate the proposed method in a proof-of-concept system with an LED illumination through a thick ground glass.

## Introduction

It is known that photons emerging from a thick disordered medium undergo multiple scattering^1^, prohibiting the image of objects behind or inside the scattering medium to form. Over the years, many approaches have been proposed to overcome this practical problem for a wide range of applications^2–4^. The most straightforward idea is to separate the ballistic photons from the scattered ones^5–8^. There are various gating techniques based on different properties possessed by these two types of photons, including their time of flight, coherence, and propagation direction, etc. Ballistic photon detection can have a high resolving power while suffering from a shallow working depth because of the exponential attenuation with respect to the penetration depth. Recent advances in wavefront shaping make it possible to measure and control the propagation of light through the scattering media^9–13^, providing an outstanding method for imaging through turbid media. One can also utilize the correlation of the speckle^14,15^. By using the angular memory effect Bertolotti *et al*. have demonstrated non-invasive imaging of fluorescent targets hidden behind a scattering layer^16^. This requires a long time of angular scanning acquisition so that it can be applicable only to relatively static targets and scattering samples. Inspired by the concept of “stellar speckle interferometry” and the angular “memory effect” for speckle correlations, Katz *et al*. have experimentally demonstrated that the target image can be reconstructed from the autocorrelation of a single-shot high-resolution scattered pattern via an iterative Fienup-type algorithm^17^. However, the iterative phase retrieval algorithm^18,19^ usually requires a large number of iterations. Moreover, such an ill-posed problem always leads to a high possibility of uncertainty^20^. To avoid iterative algorithms and eliminate the ambiguousness, the object’s Fourier phase and amplitude can be recovered via the bispectrum analysis of a single speckle pattern at the cost of the bispectrum computation burden^21,22^.

Until now, researchers mainly focus on three types of imaging systems in transillumination geometry: targets hidden behind or embedded within a scattering layer, objects hidden between two scattering layers, and imaging around the corner. However, in many cases, including label-free biological imaging, it is unlikely for a light source to exist behind the turbid medium. In addition, the targets to be observed often act as reflectors rather than emitters. Thus, for the purpose of non-invasive imaging, it is necessary to exploit imaging in reflection geometry with the light source located at the same side with the camera with respect to the scattering medium. We note that Bertolotti *et al*. have studied such a reflection geometry in imaging of labeled fluorescent object behind a scattering layer^16^. In their case the wavelength of the fluorescence light differs from the stimulated illumination, which is relatively easy to separate them. In a more general case, however, that the wavelength of the reflected light from the target is the same as the illumination, it becomes difficult to tell the difference. As a consequence, back-scattered light from the medium becomes additive noise, which is so strong that it will completely submerge the signal that is reflected from the target. Thus, a phase retrieval algorithm and bispectrum analysis will severely suffer from noise and very likely result in false reconstruction. Thus a more robust and steady technique, which is less sensitive to experimental noise, is highly in need.

In this manuscript, we propose a method of imaging reflective targets hidden behind a scattering layer with the auxiliary of a known reference object. The proposed method does not rely on the phase retrieval algorithm. Instead, it resorts to an *a priori* reference object in the neighborhood of the target. Because of the inherent connection between speckle patterns, the knowledge of an *a priori* reference object beside the target makes it possible to directly reconstruct the target image using autocorrelation or deconvolution. Compared to the phase retrieval algorithm, our method requires less computational resource and is less sensitive to noise. As a proof of the concept, we experimentally demonstrate that our method has good performance even under extremely difficult conditions that the phase retrieval algorithms may not work well.

## Theoretical Basis

### Memory effect

Because our method is based on the memory effect, it is better to address this phenomenon first. The angular range of memory effect of a scattering medium with the optical thickness (in terms of scattering mean free path) of *L* can be written approximately as^17^ $\delta \theta =\frac{\lambda }{\pi L}$, where *λ* is the wavelength of the incident light. When the object lies within this angular range, the wavelets emitted from every point of the object have approximately equal phase increments, resulting in highly correlated, but shifted, speckle patterns. The correlation properties predict a spatially invariant point spread function. For spatially incoherent illumination, all these speckle patterns are superimposed without interfering with each other. In other words, the camera image is simply a superposition of these speckle patterns, which is equivalent to the convolution of the object intensity and point spread function (PSF), namely, $I({\bf{v}})=O({\bf{u}})\ast S(\theta )$, where **u** = *l*_*u*_ × *θ* and **v** = *l*_*v*_ × *θ* indicate two-dimensional coordinates corresponding to the object plane and the imaging plane, and *l*_*u*_ is the distance between the object plane and the scattering layer, *l*_*v*_ is the distance between the scattering layer and the camera. In contrast with the conventional diffraction-limited imaging system, when an incoherent wave from the object propagates through the scattering layer, direct imaging of a hidden object is impossible because of the random nature of the speckle pattern. However, the autocorrelation of the camera image reveals that

1 $$I\otimes I=(O\otimes O)\ast (S\otimes S)\approx O\otimes O,$$

where $\otimes$ denotes the autocorrelation operation and  $\ast$ , the convolution, as a consequence of the fact that the autocorrelation of the speckle pattern is a sharply peaked function^23^. This means that the autocorrelation of the object is approximately equal to the that of the camera image. According to the Wiener-Khinchin theorem, the Fourier amplitude of the object can be deduced from its autocorrelation. From that, the object can be reconstructed using a phase retrieval algorithm or bispectrum analysis.

### The proposed method

Because the phase retrieval algorithm and bispectrum are sensitive to noise, the reconstructed image they produce tends to suffer from severe degradation. However, we found that the situation can be significantly improved if there are *a priori* objects in the neighborhood of the target of interest, as one can use them as references^24,25^. Suppose that the target, *O*(**u**), is located at a position that is laterally separated from a reference object, *R*(**u**), with the separation *δ***u** within the range of memory effect of the scattering medium *l*_*u*_*δθ*. Without loss of generality, let us denote the point response of the system to the target and the reference as *S*_1_(**u**,**v**) and *S*_2_(**u**,**v**), respectively, and the whole scene function as *O*′ = *O*(**u**) + *R*(**u** + *δ***u**). Then the camera image can be expressed as $I({\bf{v}})=O({\bf{u}})\ast {S}_{1}({\bf{u}},{\bf{v}})+R({\bf{u}}+\delta {\bf{u}})\ast {S}_{2}({\bf{u}},{\bf{v}})$ as a result of the incoherent intensity superposition. The autocorrelation of the camera image, therefore, can be written as follows:

2 $$\begin{array}{rcl}I({\bf{v}})\otimes I({\bf{v}}) & = & O({\bf{u}})\otimes O({\bf{u}})+R({\bf{u}}+\delta {\bf{u}})\otimes R({\bf{u}}+\delta {\bf{u}})\\  &  & +O({\bf{u}})\otimes R({\bf{u}}+\delta {\bf{u}})\ast ({S}_{1}\otimes {S}_{2})\\  &  & +R({\bf{u}}+\delta {\bf{u}})\otimes O({\bf{u}})\ast ({S}_{2}\otimes {S}_{1}),\end{array}$$

Equation (2) suggests that there are four terms in the autocorrelation of the camera image. The first two terms consist of the autocorrelation of the target and the autocorrelation of the reference, and they are located at the center. Here we are more interested in the interaction between *O*(**u**) and *R*(**u** + *δ***u**). Taking advantage of the fact that the reference object is known and amplitude-only, we can further process the cross-correlation terms for subsequent reconstruction:

3 $$\begin{array}{rcl}O({\bf{u}})\otimes R({\bf{u}}+\delta {\bf{u}})\ast ({S}_{1}\otimes {S}_{2}) & = & O(\,-\,{\bf{u}})\ast R({\bf{u}}+\delta {\bf{u}})\ast ({S}_{1}\otimes {S}_{2}),\\ R({\bf{u}}+\delta {\bf{u}})\otimes O({\bf{u}})\ast ({S}_{2}\otimes {S}_{1}) & = & O(\,-\,{\bf{u}})\ast R({\bf{u}}-\delta {\bf{u}})\ast ({S}_{2}\otimes {S}_{1}),\end{array}$$

clearly, the third term in Eq. (2) indicates the convolution of the laterally shifted target and the reference object, whereas the forth gives a symmetrically shifted convolution. The displacement of the convolution terms results from the relative shift *δ***u**. Because *S*_1_ and *S*_2_ are both speckle patterns, it can be deduced that ${S}_{1}\otimes {S}_{2}$ and ${S}_{2}\otimes {S}_{1}$ are sharply peaked functions weighted by their correlation coefficient^26^, denoted as *γ*. When the target and reference objects are significantly separated from each other, the system response to them may be statistically uncorrelated. As a result, ${S}_{1}\otimes {S}_{2}$ and ${S}_{2}\otimes {S}_{1}$ equal to 0. In this case, the convolution terms in Eq. (2) will disappear. Thus, it is necessary for the target and reference object to fall within the range of memory effect of the scattering medium, which imposes an upper bound on the allowable lateral shift *δ***u**. In an extreme case that the reference is a point object with the size negligible with respect to the target, the convolution term is approximately equal to target. Similar to the work done by Singh *et al*.^27^, image reconstruction can be accomplished by calculating the cross-correlation between the camera image and the point spread function, except that our method does not require the operation in the time domain at the cost of introducing a reference point in space. By ensuring that the convolution terms are separated from the central autocorrelation, it is easy to directly window out the final reconstructed object from the speckle autocorrelation. As a rule of thumb, the distance between the reference point and target object should be at least 1.5 times larger than the size of the target.

In a more general case, the size of the reference object cannot be neglected, and the shape may be irregular. Therefore, direct recognition of the target from the autocorrelation of the camera image is usually impossible. The image reconstruction should be realized by further deconvolution. From the autocorrelation of the camera image, we have $O({\bf{v}})\ast R({\bf{v}})$. Thus, the magnification factor must be taken into consideration during calculation. However, practically, we found that direct deconvolution is still difficult and unprofitable. First, as mentioned above^26^, ${S}_{1}\otimes {S}_{2}$ is a sharply peaked function weighted by the factor *γ*. The convolution terms then are weighted by the same factor as well. Second, background noise severely distorts the convolution terms. Further processing of the speckle autocorrelation may lead to a false result owing to the ill-poseness of the problem^28^. Thus, direct deconvolution using only the known *R*(**u**) is shown to be unreasonable. If, however, there is a way to record the speckle pattern of the reference object first, and then record a speckle pattern of the target together with the reference object in the same scene, the problem becomes easier to solve. The target object can be reconstructed with high fidelity using the convolution theorem:

4 $$O({\bf{u}})={ {\mathcal F} }^{-1}\{\frac{ {\mathcal F} \{{I}_{O}\}}{ {\mathcal F} \{{I}_{R}\}}\times  {\mathcal F} \{R(M{\bf{u}})\}\},$$

where *I*_*R*_ and *I*_*O*_ are the speckle patterns deriving from the reference and the target, independently. ${\mathcal F}$ and ${ {\mathcal F} }^{-1}$ correspond to the Fourier and inverse Fourier transform, respectively, and the magnification factor $M=\frac{{l}_{v}}{{l}_{u}}$.

In contrast with the memory-effect-based deconvolution microscopy proposed by Edrei^29^, we use a relatively large object rather than a small iris to characterize the scattering layer because a small iris develops a relatively feeble speckle pattern in comparison to the strong backscattered noise. In addition, in Edrei’s work^29^, the object distance *S*_*o*_ and the image distance *S*_*I*_ must satisfy the thin lens formula. The thin diffuser was only inserted to introduce weak disturbance. Thus, in order to design the system, one must know (or calibrate) the object distance. But this is unlikely to be possible when the scattering medium becomes optically thick. In our scheme, on the contrary, we do not need a physical lens, but treat the scattering medium itself as a scattering *lens*^15^. This means that there is no limitation to set the object distance or image distance.

## Experimental Results

### Experimental setup

**Figure 1:**
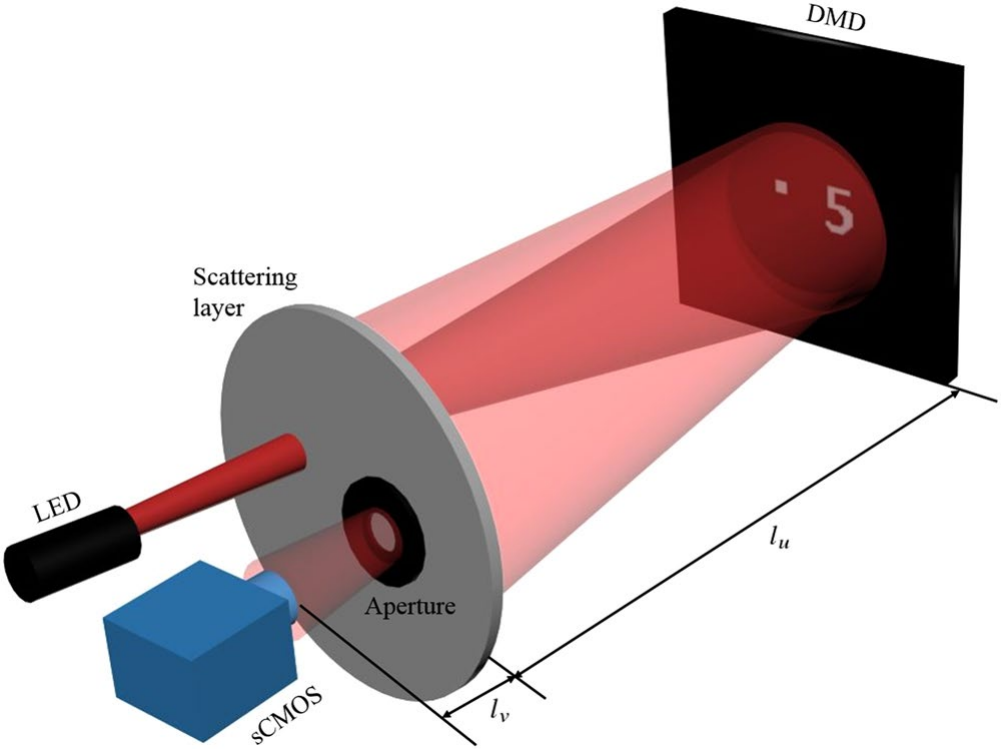
Optical setup. A spatially incoherent light source transmits through the ground glass and shone on a reflective target hidden behind it. The reflected light propagates through the ground glass in the opposite direction and was collected by an sCMOS camera.

The experimental setup is schematically shown in Fig. 1. An LED (Thorlabs’ mounted high-power LED M625L3) with the central wavelength of 625 nm and the bandwidth of 16 nm was used as an incoherent illumination source. The LED beam was shined onto the surface of the scattering medium, which was a ground glass in our case with effective scattering thickness measured to be equaling to 35.757 *μ*m^14^. Backscattered waves from the ground glass caused strong background noise, which was detrimental to our experimental results. A forward scattering beam impinged on the target plane, which was placed 60 cm away from the ground glass. For the sake of convenience, the object was a pattern displayed on a digital micro-mirror device (DMD) with the pixel size of 10.8 *μ*m × 10.8 *μ*m. To guarantee that the object was within the range of memory effect of the ground glass, the object image written on the DMD had the dimension of 0.7 to 3 mm. The light reflected from the object was obliquely retransmitted through the ground glass and then collected by an sCMOS camera (PCO Edge 4.2). The inclination angle was measured to be 0.215 rad. Under this circumstance, the half width at half maximum angle, namely, the field of view of the memory effect was measured to be 0.006 rad^14^. An aperture was placed against the ground glass to select a suitable diffraction order resulting from the pixilation of the DMD.

To satisfy the Shannon-Nyquist sampling condition, a single speckle grain should be twice larger than the camera pixel pitch. The average size of the speckle grain can be calculated by *λl*_*v*_/*D*, where *D* denotes the size of the aperture against the ground glass^17^. The sampling constraint could be met by adjusting either the distance or the size of the aperture. In our experiments, the sCMOS camera was placed approximately 15 cm from the ground glass on the other side, and the diameter of the aperture was approximately 3 mm.

### Results

**Figure 2:**
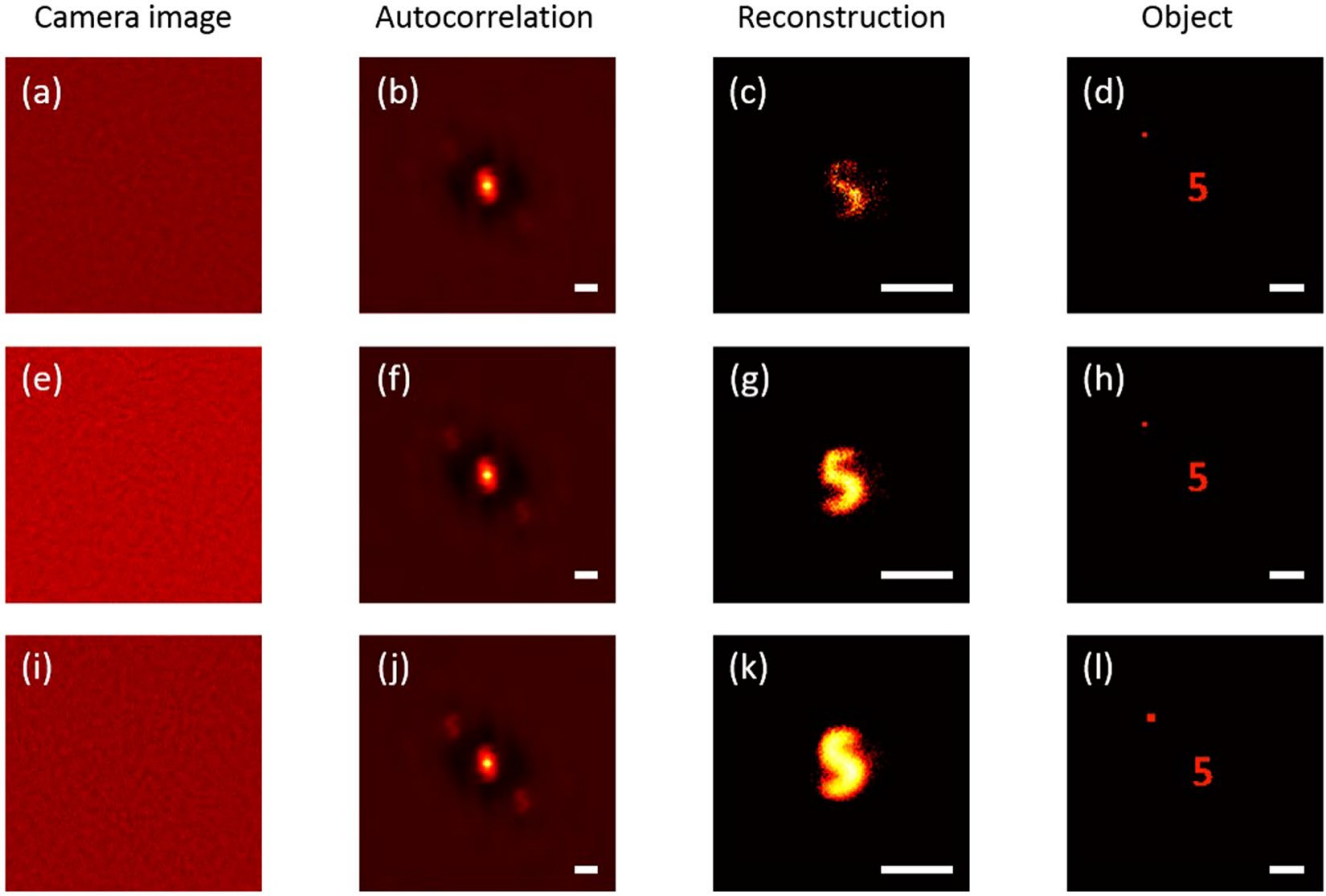
Experimental results: Reconstruction with a reference point. (**a**) Raw camera image after spatial normalization. (**b**) Corresponding autocorrelation of the camera image. (**c**) Object reconstructed from autocorrelation of (**b**) by direct selection. (**d**) Original object containing the target object and reference point. (**e**–**l**), As in (**a–d**) for reference points with different sizes. Scale bars: 1 mm at the object plane.

In the first experiment, we considered a small reference point with a size far smaller than the target object, which was the digit “5” with the size of approximately 700 *μ*m in the object plane. The distance between the reference point and the target center was 2 mm. The correlation coefficient was measured to be about 0.60. Let us first examine the case that the size of the reference point is 10 × 10 pixels. The experimental results are plotted in Fig. 2. The speckle pattern captured by the camera is usually modulated by a slowly varying envelope due to the uneven illumination. To remove this envelope, one can normalized it by using a low-pass filtered version of it. The resulting speckle are shown in Fig. 2(a). Taking the ergodicity of the scattering process into consideration, we can calculate the autocorrelation of the target from the speckle pattern by replacing the temporal ensemble average with the spatial average. Instead of calculating the autocorrelation of many single-shot speckle patterns, we divided a single-shot image into many sub-images with 500 × 500 pixels in size, calculated the autocorrelation of each sub-image, added these results together, and then calculated the mean^30^. In the process, the sharply peaked function was enhanced and the noise was balanced out, leading to the suppression of random noise, as shown in Fig. 2(b). One can clearly see a bright spot at the center and two other dimmed spots at two symmetric positions with respect to the centre. The central corresponds to the first two terms in Eq. (2), whereas the two dimmed spots correspond to the cross terms. we can then select one of the cross terms, and binarize it, yielding the reconstructed image as shown in Fig. 2(c). Comparing with the ground-truth target plotted in Fig. 2(d), one can clearly see that the digit “5” has been reconstructed, but the contrast is low owing to the observation that the background noise overwhelms the convolution term as suggested by Fig. 2(b).

When we increased the size of the reference point to be 15 × 15 pixels, and repeated the aforementioned reconstruction process, we obtained the reconstructed image shown in Fig. 2(g). It is clearly seen that this image is much better than the one shown in Fig. 2(c) in terms of brightness, contrast and feature details. However, when we continued to increase the size of the reference point, for example, to 20 × 20 pixels, the reconstructed image becomes vague because the convolution with a square-like point degrades the resolution, as shown in Fig. 2(k). In practical applications, if the size of the reference point is adjustable, one should choose an appropriate one. If it is too small, the reconstruction will suffer from severe background distortion; if it is too large, the reconstruction undergoes resolution degradation.

**Figure 3:**
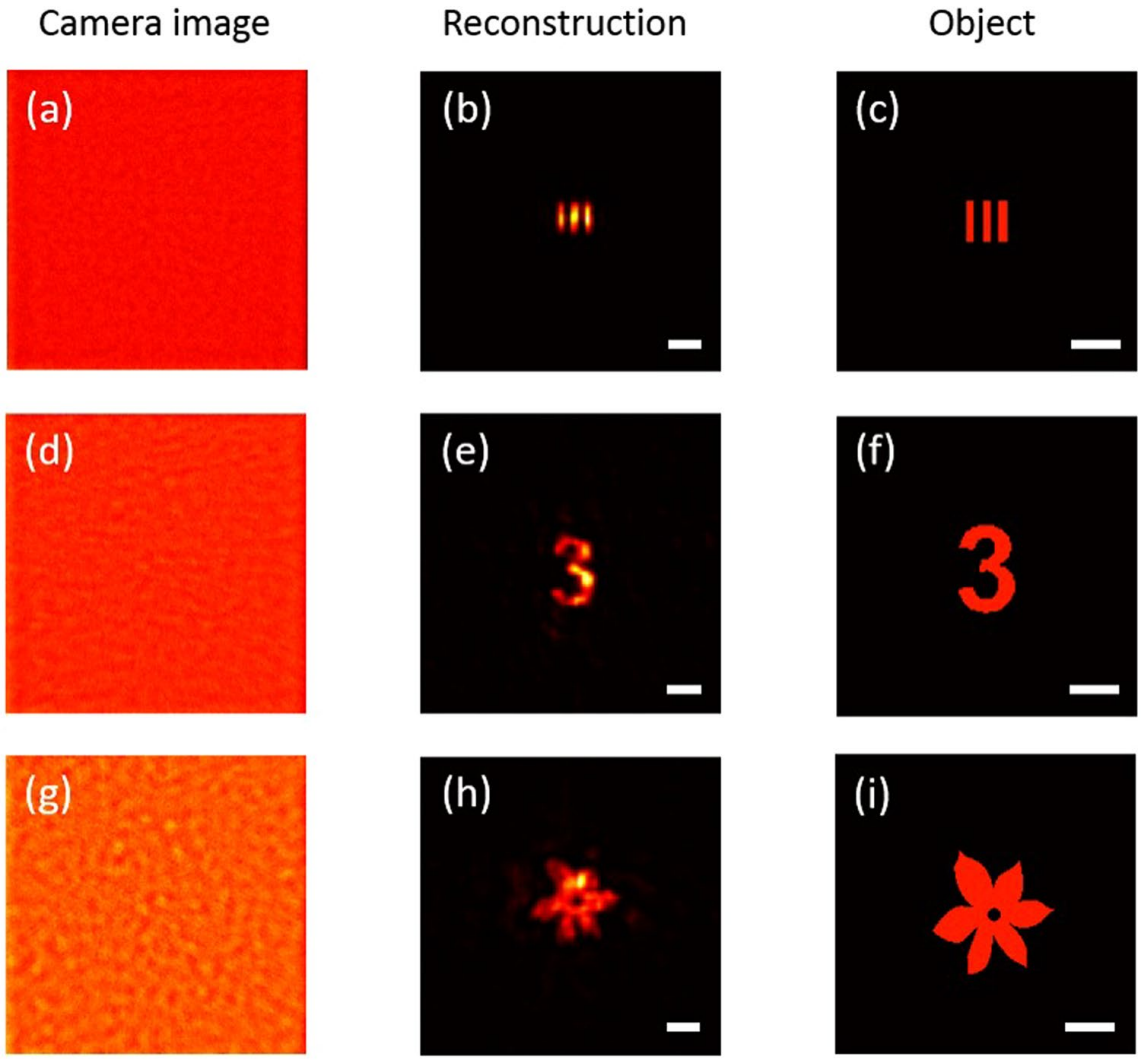
Deconvolution with reference object. (**a**) Raw camera image after spatial normalization. (**b**) Object reconstructed from the camera image. (**c**) Original object. (**d**–**i**), As in (**a–c**) for different objects. Scale bars: 1 mm at the object plane.

In many cases, however, the reference object is not selectable. Deconvolution is then necessary to reconstruct an object. For the sake of demonstration, we simply used the digit “5” in the previous experiment as the reference object in our study, and the corresponding speckles collected by the camera acted as the reference speckle. Then, we replaced it with different targets and collected the corresponding speckle patterns, the normalized of which are shown in Fig. 3(a,d,g). The image reconstruction was calculated from the speckle patterns via Eq. (4). Again, when calculating the autocorrelation, we used the ergodic property of the speckle to suppress random noise^30^. We divided the reference speckle and the target speckle patterns into sub-images of 500 × 500 pixels in size, and used Eq. (4) to conduct the deconvolution for each sub-image and obtained a reconstructed images, which are noisy as expected. But the statistical averaging of all these reconstructed sub-images, the signal-to-noise ratio can be dramatically improved as shown in Fig. 3(b,e,h). It is not difficult to find that the final reconstructed images retained all the features of the corresponding targets, including the spacing between the vertical stripes in the “bar”, the asymmetry of the digit “3”, and the six petals in the “flower”, indicating its superiority in reconstructing objects with high fidelity. Indeed, we have calculated the correlation coefficient between the reconstructed images and the corresponding ground-truth target images shown in Fig. 3(c,f,i), and found that the correlation coefficients were 0.846, 0.892, and 0.864, respectively.

Although the reference point and reference object deconvolution corresponded to different processing methods, the inherent nature is the same. The speckle arising from the reference point or reference object should be correlated with those arising from the target, so that the system responses to them are correlated. The reference point is just a special case of the object profile; while the reference object deconvolution is a more general method, which provides a high fidelity reconstruction.

**Figure 4:**
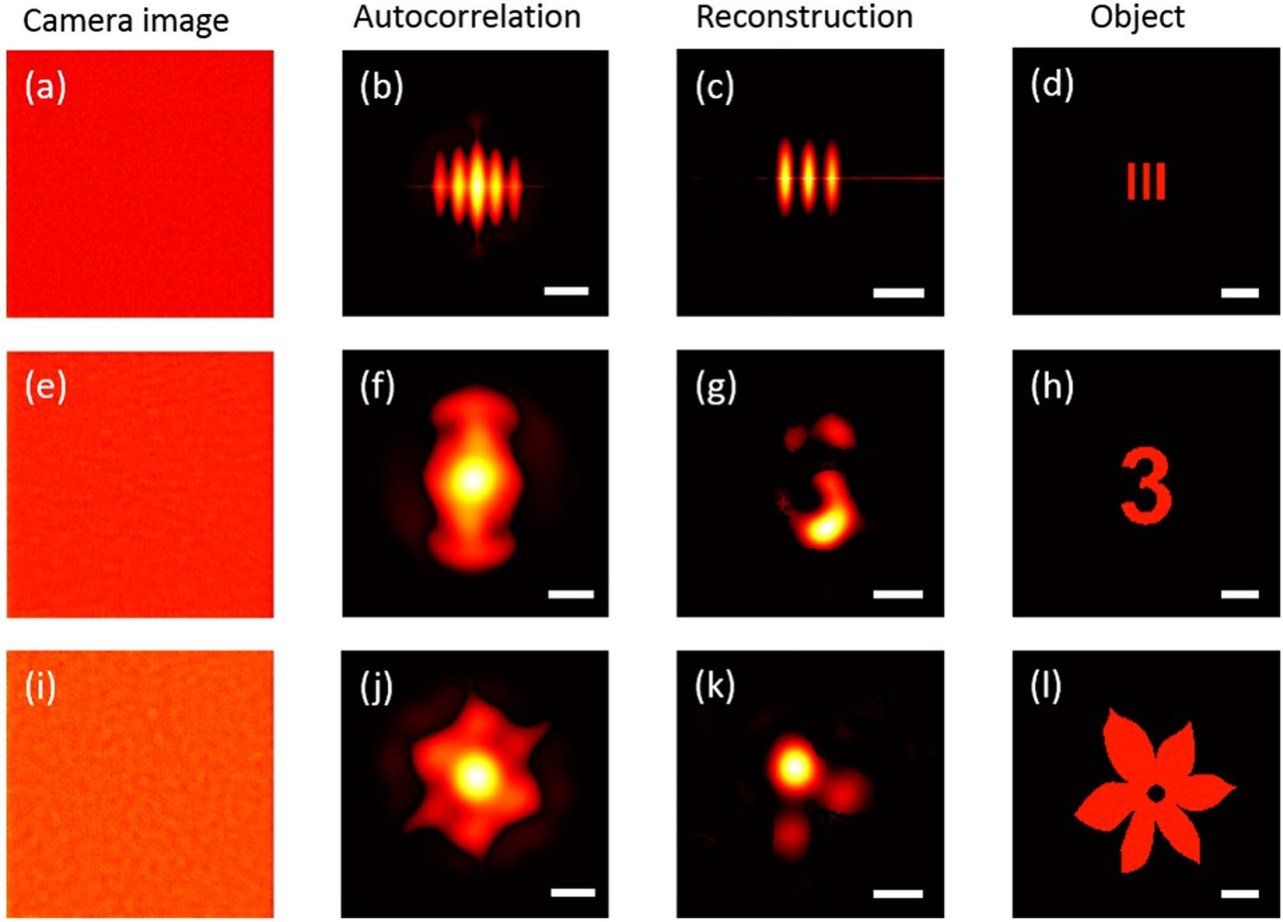
Reconstruction with phase retrieval algorithm. (**a**) Raw camera image after spatial normalization. (**b**) Autocorrelation of the camera image. (**c**) Object reconstructed with phase retrieval algorithm. (**d**) Original object. (**e**–**l**), As in (**a–c**) for different objects. Scale bars: 1 mm at the object plane.

To demonstrate the superiority of our method, we make a comparison of our results with those reconstructed using the phase retrieval algorithm, and plot them in Fig. 4. Figure 4(a,e,i) show the speckle patterns of the target ‘bar’, digit ‘3’ and ‘flower’ with the slowly varying envelope eliminated, respectively. We divided the camera image into multiple sub-images and added the autocorrelations together to generate a more accurate autocorrelation, which are shown in Fig. 4(b,f,j). Because the phase retrieval algorithm mostly produce approximate solutions and highly dependent on the initial condition, we used this algorithm about 30 times with different initialization^20^, and the best reconstructed images were plotted in Fig. 4(c,g,k). It is clearly seen that the “bar” can be well reconstructed, while the digit “3” and the “flower” become distorted and patchy. A strong background usually brings about a noisy autocorrelation, which leads to failure reconstruction. It is obvious that the phase retrieval algorithm is more sensitive to noise than our method.

## Conclusion

In conclusion, we have demonstrated a novel technique for imaging behind a scattering layer in a reflective geometry. The proposed technique does not rely on phase retrieval algorithms or bispectrum analysis, but use the *a priori* information about a reference point or reference object in the neighborhood of the target for image reconstruction. It has been shown that the knowledge of a reference point can help to determine the outline of the target quite easily. One just needs to take the autocorrelation of the captured speckle pattern, which directly reveals the target image. In the general case that the reference is not a point, but an arbitrary known object, deconvolution method should be employed to generate a high-fidelity reconstruction.

According to our study, there are several conditions for the proposed method to have better performance. First, because our method is based on the memory effect, the object should fall within the angular range of the memory effect. It is important to note that the reference point and the target should be separated from each other with a certain distance so that the cross-correlation terms and the autocorrelation terms can be separated. Appropriate separation should be taken into account in the deconvolution method for a general reference object. Or the system response to the reference object and target will not correlate. Second, spatially incoherent light coming from the LED as in our experiments is scattered when it hits the ground glass in the first place. This yields a strong but low contrast speckle pattern, which acts as a strong backscattered noise and severely degrades the speckle pattern produced by the target. As a consequence, the signal-to-noise ratio of the final reconstructed image is significantly reduced, or even completely submerged in noise. This should be taken into account when designing the system. Further investigation should be made to reduce the influence of the backscattered light. Third, in many practical applications, the light reflected from the object is obliquely incident on the scattering layer. The oblique angle leads to an increase in the scattering medium’s effective thickness, making the range of memory effect much smaller than the theoretical prediction^14^. The part of the target that is beyond the range of memory effect will not generate correlated speckle, yielding additional noise to the reconstructed image, and reducing the field of view. Forth, as wavelength influences the size of speckle grains, illuminating by broad bandwidth source will give rise to fuzzy speckle patterns^31^ and increase background level in the speckle autocorrelation function. This means that better reconstruction results can be obtained with narrower bandwidth illumination. Although we demonstrated our method with a plane object, it applies equally to 3D objects. We can realize 3D object imaging by taking advantage of the longitudinal memory effect, as long as the objects also fall within the axial memory effect range. By varying the longitudinal position of the reference object, we can focus on objects and realize imaging at different depths. In addition, as our method just needs one single-shot speckle to reconstruct the target image when the *a priori* information is reference point. In this case, it is in principle possible to implement in real time^17,32^. But the detailed discussion on the real time implementation is out of the scope of this manuscript.
